# SIX-MONTH SUPERVISED TELEREHABILITATION COMBINING PILATES-BASED THERAPEUTIC EXERCISE AND SELF-MYOFASCIAL RELEASE IN ADULTS WITH FIBROMYALGIA: A RANDOMIZED CONTROLLED TRIAL

**DOI:** 10.2340/jrm.v58.46786

**Published:** 2026-10-13

**Authors:** Thomai MARANTIDOU, Evangelia I. GERMANOU, Vasiliki MANOU, Nikolaos KOUTLIANOS, Evangelos SYKARAS, Ioannis TERZIDIS

**Affiliations:** 1 Department of Physical Education and Sport Science, Aristotle University of Thessaloniki, Thessaloniki; 2 TheMIS Orthopaedic Center, Thessaloniki, Greece

**Keywords:** fibromyalgia, Pilates, rehabilitation, self-myofascial release, telerehabilitation, therapeutic exercise

## Abstract

**Objective:**

To evaluate the effects of a 6-month supervised multicomponent telerehabilitation programme combining Pilates-based therapeutic exercise and self-myofascial release on fibromyalgia impact, sleep quality, and psychological outcomes.

**Design:**

Two-arm, parallel-group randomized controlled trial.

**Subjects/Patients:**

68 adults with fibromyalgia confirmed according to the 2016 ACR criteria.

**Methods:**

Participants were randomized to a 6-month supervised home-based telerehabilitation programme (*n* = 35) or usual care (*n* = 33). The intervention comprised 3 live online sessions weekly combining Pilates-based therapeutic exercise, self-myofascial release, core stabilization, low-load resistance exercise, stretching, and relaxation. Outcomes were assessed at baseline, 3 months, and 6 months using validated patient-reported measures.

**Results:**

Significant group-by-time interactions were observed for fibromyalgia impact (*p* < 0.001, partial η^2^ = 0.132), sleep quality (*p* < 0.001, partial η^2^ = 0.129), depressive symptoms (*p* = 0.002, partial η^2^ = 0.129), and perceived stress (*p* = 0.028, partial η^2^ = 0.065), but not for anxiety (*p* = 0.701). Mean intervention adherence was 87.5%, with no adverse or serious adverse events reported in either group.

**Conclusion:**

6 months of supervised multicomponent telerehabilitation produced favourable longitudinal changes in fibromyalgia impact, sleep quality, depressive symptoms, and perceived stress compared with usual care.

## LAY ABSTRACT

Fibromyalgia is a long-term condition that can affect pain, sleep, daily activities, and emotional well-being. This study investigated whether a 6-month multicomponent telerehabilitation programme could help adults with fibromyalgia. Sixty-eight patients were assigned either to the rehabilitation programme or to continue their usual daily activities. The programme was performed at home 3 times per week, primarily through live supervised online sessions, and combined Pilates-based exercise, self-myofascial release, strengthening, stretching, and relaxation. Participants receiving the programme showed greater improvements over time in the overall impact of fibromyalgia, sleep quality, depressive symptoms, and stress than those receiving usual care. Anxiety did not differ between groups. Programme adherence was high, and no adverse events or serious adverse events were reported in either group. These findings suggest that supervised online rehabilitation may provide a practical and effective way of delivering long-term exercise-based care to people with fibromyalgia.

Fibromyalgia (FM) is recognized as a nociplastic pain condition driven primarily by central sensitization mechanisms and clinically characterized by generalized and persistent musculoskeletal pain for more than 3 months (1). Peripheral myofascial mechanisms may also contribute to the clinical expression of widespread pain and mechanical hypersensitivity (2). Beyond pain, FM encompasses multiple interacting symptoms including sleep disturbance and chronic fatigue, as well as impaired physical functioning and psychological distress, which collectively contribute to reduced health-related quality of life (3–5).

This multidimensional symptomatology necessitates a holistic approach to therapeutic management. Contemporary guidelines emphasize non-pharmacological treatment, with exercise representing a central component, alongside relaxation techniques and interventions aimed at improving sleep and mental health (6). In this context, Pilates-based protocols have shown potential benefits for pain, physical function, and overall well-being in FM patients (7, 8). A recent systematic review and meta-analysis of 7 randomized controlled trials found that Pilates-based interventions did not demonstrate superiority over other active exercise modalities for pain or FM impact, although interpretation was limited by low-to-very-low certainty of evidence, heterogeneous protocols, and the absence of pooled long-term data (9). These findings support Pilates as a potentially useful exercise option within multimodal FM rehabilitation rather than as a demonstrably superior modality.

Myofascial release offers another promising therapeutic pathway (10). Self-myofascial release (SMR), including foam rolling, elicits acute gains in joint range of motion and pain attenuation while preserving neuromuscular performance (11, 12). In individuals with FM, these interventions also positively impact sleep and emotional well-being (13–17). Yet, clinical trials evaluating combined Pilates and SMR protocols remain scarce, pointing to a critical area for future investigation.

This need for multicomponent care is further amplified by shifts in how rehabilitation is administered. Telerehabilitation offers a practical framework for extending therapy while mitigating geographical and logistical barriers. Existing data support its clinical value in FM management (18), with recent randomized trials validating virtual Pilates delivered through synchronous or asynchronous digital health models (19, 20). However, outcomes typically depend on intervention design, specific protocols, and degree of clinician supervision (21). Although evidence supports telerehabilitation for FM and myofascial interventions have been evaluated separately in this population, there is still a scarcity of data regarding long-term, supervised telerehabilitation programmes integrating therapeutic exercise with SMR techniques (10, 17–21).

To bridge these gaps, this randomized controlled trial (RCT) aimed to evaluate the effects of a 6-month, supervised multicomponent telerehabilitation programme combining Pilates-based therapeutic exercise and SMR on overall FM impact. Secondary objectives included assessing efficacy across sleep quality and psychological domains – specifically depressive symptoms, anxiety, and perceived stress – while exploring clinical outcome associations throughout the intervention. We hypothesized that this programme would yield superior improvements in disease impact, sleep, and psychological distress compared with usual care. Furthermore, we anticipated that reductions in overall disease impact would correlate with enhanced sleep quality and psychological well-being.

## METHODS

### Study design

This two-arm, parallel-group, superiority randomized controlled trial evaluated a 6-month home-based multicomponent telerehabilitation programme in patients with FM. Outcomes were assessed at baseline (T0), 3 months (T1), and 6 months (T2). The intervention group received a supervised, synchronous telerehabilitation combining Pilates-based therapeutic exercise with SMR, whereas the control group maintained their usual daily activities without initiating a new structured exercise programme.

The study was carried out in accordance with the Declaration of Helsinki and was approved by the Ethics and Research Integrity Committee of the Department of Physical Education and Sport Science, Aristotle University of Thessaloniki (approval no. 201/2024; 12 November 2024). All participants provided written informed consent prior to participation. Participant enrolment took place from 6 to 20 January 2025 and final study data collection was completed on 10 August 2025. The trial was retrospectively registered with the ISRCTN registry (ISRCTN18189260) on 20 August 2026. No patients, patient representatives, or members of the public were involved as research partners in the design, conduct, analysis, or reporting of the trial. No important changes to the trial methods or outcomes were made after trial commencement.

### Participants

Participants were recruited remotely through electronic invitations posted in FM patient-support groups on social media. Of 72 individuals who expressed interest in participating during the recruitment period, 4 were excluded before randomization: 3 following medical eligibility screening by the study physician (sarcoidosis, rheumatoid arthritis, and uncontrolled hypertension, respectively) and 1 because of inability to use the videoconferencing platform, leaving 68 participants for baseline assessment and randomization (Fig. S1). Baseline demographic and medical-history information was collected using a self-administered electronic questionnaire completed by participants before randomization. Other medical-history variables were self-reported and were not independently verified against medical records or laboratory reports. An *a priori* sample size calculation (power analysis) was performed using G*Power version 3.1.9.7 (https://www.psychologie.hhu.de/arbeitsgruppen/allgemeine-psychologie-und-arbeitspsychologie/gpower), based on the primary outcome of overall FM impact assessed using the Revised Fibromyalgia Impact Questionnaire (FIQR) at the 6-month assessment. Based on previous FM trials, a target between-group difference of 8.1 FIQR points was assumed, corresponding to an estimated clinically important difference derived from the original FIQ (22). A common standard deviation of 11.5 points was assumed, corresponding to a standardized effect size of Cohen’s *d* = 0.70. With a two-sided significance level of α = 0.05, 80% statistical power (1 – β = 0.80), and a 1:1 allocation ratio, the analysis indicated that a minimum of 33 participants per group (*N* = 66) was required. The final randomised sample comprised 68 participants (intervention, *n* = 35; control, *n* = 33), all of whom provided FIQR data through to the 6-month assessment, thereby exceeding the prespecified minimum sample-size requirement.

Potential participants who self-reported a previous diagnosis of FM underwent eligibility screening by a physician member of the research team. The physician obtained a medical history and confirmed eligibility, including the diagnosis of FM according to the 2016 American College of Rheumatology (ACR) criteria (23). Inclusion criteria were: age ≥ 18 years; FM meeting the 2016 ACR criteria; no restriction on disease duration; stable regular pharmacological treatment for at least 1 month before the baseline assessment; sedentary status; sufficient written Greek proficiency; and access to an internet-enabled device with the digital skills required for interactive videoconferencing. Exclusion criteria, assessed by the study physician during the medical-history screening, were: unstable cardiovascular or respiratory disease; coexisting systemic inflammatory/rheumatic conditions, which could confound the assessment of widespread musculoskeletal pain; uncontrolled hypertension, or severe neurological disorders precluding safe physical activity; a recent acute musculoskeletal episode or surgery compromising exercise tolerance; and cognitive or sensory impairment preventing effective remote participation or questionnaire completion.

### Randomisation and blinding

Participant enrolment was performed by the researcher responsible for recruitment, whereas medical eligibility screening was performed by a physician member of the research team. Following completion of the baseline assessment, participants were randomly allocated to the intervention or control group using a computer-generated randomization sequence, with an intended 1:1 allocation ratio. Group assignment was implemented automatically by the electronic system. The researcher responsible for participant enrolment did not have access to the allocation sequence before assignment and therefore could not foresee upcoming allocations. The resulting allocation comprised 35 participants in the intervention group and 33 in the control group.

Due to the active nature of the online supervised exercise intervention, blinding of participants and the intervention provider was not feasible. To minimize investigator-related assessment bias, outcome data were collected using self-administered electronic questionnaires. Group identity was masked during data processing and statistical analysis by coding the study arms as “Group 1” and “Group 2”. All statistical analyses were completed using these coded labels, and group identity was revealed only after completion of the final analyses.

### Intervention and control group

Participants allocated to the intervention group completed a 6-month, supervised, group-based telerehabilitation programme delivered live via videoconferencing software (Zoom; Zoom Communications, Inc, San Jose, CA, USA). The intervention was delivered by a doctoral candidate and graduate of the Department of Physical Education and Sport Science, Aristotle University of Thessaloniki, with specialization in Special Physical Education – Therapeutic Exercise, a postgraduate degree, and certification in Pilates and SMR techniques. Three 50–60 min sessions were delivered weekly, with identical morning and afternoon cohorts offered to facilitate attendance. Both daily cohorts followed the same exercise protocol, and participants who were unable to attend their usual group could attend the corresponding live session at the alternative time. When attendance at either live session was not feasible, participants were offered a recorded version of the same 50–60 min session as a make-up option. Recordings were made available through restricted Google Drive links accessible only to study participants and were intended solely as an alternative for missed live sessions, rather than as a routine mode of programme delivery. Participants completing a recorded session were required to confirm completion by direct text message to the research team; these self-reported completions were documented in the study attendance registry and included in the calculation of programme adherence.

Regarding the home-based exercise protocol, each session comprised approximately 10 min of diaphragmatic breathing, low-intensity mobility, and core activation; 10 min of mobility and SMR using foam rollers or tennis balls, emphasizing the spine and hip region; 25–30 min of mat-based Pilates, core stabilization, and low-load resistance exercise; and 5–10 min of stretching and relaxation techniques. Exercise volume, duration, movement complexity, and resistance were progressively increased using symptom-contingent rather than strictly time-contingent progression. During live sessions, participants could communicate with the intervention provider at any time and report pain, fatigue, discomfort, or difficulty with an exercise. The provider also actively checked participants’ symptoms and tolerance frequently throughout the session, typically after individual exercises or short exercise sequences. When symptoms or difficulty were reported, exercises were modified in real time by reducing the number of repetitions, allowing rest, adjusting technique or movement execution, or discontinuing the exercise when necessary. Participants were consistently encouraged to prioritize correct technique and symptom tolerance over completing a predetermined number of repetitions. Exercise equipment included a mat, foam roller, tennis balls, and simple household items as required.

A total of 72 sessions were scheduled for each participant. Live sessions, which represented the primary mode of programme delivery, were supervised synchronously. The intervention provider demonstrated movements and provided continuous verbal cues, ensuring immediate biomechanical corrections in real time. Exercises were adapted to individual symptoms and tolerance. Programme adherence was calculated as the percentage of attended sessions relative to the total scheduled sessions. This included attendance at live supervised sessions, as well as self-reported completion of recorded make-up sessions when live attendance was not possible. Live attendance was recorded directly by the research team via a digital log, whereas completion of recorded sessions was documented following written confirmation from the participant.

Control participants were asked to continue their usual daily routines without adding any new or formal exercise habits throughout the 6-month study period. Participants in both groups were permitted to continue their usual pharmacological treatment throughout the study. Regular medication use was recorded, and participants were instructed not to initiate a new structured exercise programme outside the study protocol during the intervention period.

### Adverse events and safety monitoring

Adverse events (AEs) were systematically monitored throughout the 6-month intervention period. Participants in both groups were instructed to report spontaneously any untoward medical occurrence, physical discomfort, or injury, and the research team additionally screened for AEs through frequent symptom-triggered follow-up communications, primarily via the Messenger messaging platform. When participants reported pain or increased FM-related symptoms before a session, they were encouraged to provide an update later the same day or were contacted by the intervention provider to assess whether symptoms had improved, persisted, or worsened after exercise. Reported events were recorded and evaluated by the principal investigator according to severity (mild, moderate, or severe) and their relationship to the telerehabilitation programme (not related, possibly related, or definitely related). Particular attention was given to potential exercise-related events, including transient muscle soreness, joint pain, and exacerbation of fibromyalgia symptoms. Serious adverse events (SAEs) were defined as events resulting in hospitalization, prolonged disability, or a life-threatening condition and required immediate reporting.

### Outcome measures

Outcomes were collected remotely using self-administered electronic questionnaires at T0, T1, and T2. Personalized e-mail notifications containing secure survey links were used for follow-up assessments. Contact information was stored separately from questionnaire responses and was accessible only for longitudinal follow-up.

The primary outcome was overall FM impact, quantified via the Revised Fibromyalgia Impact Questionnaire (FIQR) (24). The 21-item FIQR assesses physical function, overall impact, and symptom severity (0–100), with higher scores indicating greater disease impact. As no validated Greek version was available at the time of study conception, the FIQR was translated and culturally adapted into Greek using forward- and back-translation followed by an expert-panel review and cognitive debriefing in 10 Greek-speaking individuals with FM, following established cross-cultural adaptation principles (25). The resulting version underwent a pilot psychometric evaluation in an independent sample of 31 adults with FM, demonstrating excellent internal consistency for the total scale (Cronbach’s α = 0.922) and satisfactory-to-excellent internal consistency across its domains (α = 0.717–0.908). These psychometric data have not previously been published.

Secondary outcomes were sleep quality, assessed using the Pittsburgh Sleep Quality Index (PSQI) (26), and depressive symptoms, anxiety, and perceived stress, assessed using the Depression Anxiety Stress Scales-42 (DASS-42) (27). Τhe PSQI comprises 19 self-rated items generating 7 component scores and a global score ranging from 0 to 21, with higher values reflecting poorer sleep quality. The DASS-42 consists of 42 items divided into 3 x 14-item subscales evaluating depression, anxiety, and perceived stress; higher subscale scores indicate greater symptom severity.

While FIQR and PSQI data were complete for all 68 participants across all time points, DASS-42 questionnaire non-response occurred exclusively in the intervention group: 8 participants had missing assessments at 3 months and 13 at 6 months. These missing assessments reflected questionnaire non-response rather than study withdrawal. Longitudinal DASS-42 analyses therefore used complete cases across all 3 assessment points (*n* = 55; intervention, *n* = 22; control, *n* = 33).

### Statistical analysis

Statistical analyses were performed using IBM SPSS Statistics version 29.0 (IBM Corp, Armonk, NY, USA). Descriptive statistics were used to summarize participants’ demographic and clinical characteristics. Continuous variables are presented as mean and standard deviation (SD), and categorical variables as frequencies and percentages. Outcome scores used in non-parametric between-group comparisons are additionally presented as medians (Q1–Q3). Internal consistency was assessed using Cronbach’s alpha. Between-group comparisons at each time point were performed using Mann–Whitney *U* tests. Supplementary unadjusted between-group mean differences (intervention minus control) with 95% confidence intervals were calculated at the 3- and 6-month assessments. These estimates complemented the Mann–Whitney *U* tests and longitudinal mixed-ANOVA analyses. Longitudinal effects were examined using mixed repeated-measures analysis of variance with time (T0, T1, T2) as the within-subject factor and group (intervention, control) as the between-subject factor. Normality and sphericity assumptions were assessed, with Greenhouse–Geisser correction applied when appropriate. Effect sizes are reported as partial eta squared (η^2^p). FIQR and PSQI analyses included all 68 participants, whereas DASS-42 included 55 complete cases. Exploratory associations among FIQR, PSQI, depression, anxiety, and stress scores at each assessment time point were examined using Spearman’s rank correlations with pairwise available observations. Statistical significance was set at *p* < 0.05.

## RESULTS

### Participant characteristics and study data

Baseline demographic and clinical characteristics are presented in Table I. A total of 68 participants were randomized to the intervention (*n* = 35) or control group (*n* = 33). Participants were predominantly women (97.1%); most were aged 41–60 years and had been diagnosed with FM within the previous 1–6 years (58.9%).

**Table I:** Baseline demographic and clinical characteristics of the participants

Characteristic	Intervention (*n* = 35)	Control (*n* = 33)	Total (*N* = 68)
Female sex, *n* (%)	34 (97.1)	32 (97.0)	66 (97.1)
Age group, *n* (%)			
18–30 years	0 (0.0)	1 (3.0)	1 (1.5)
31–40 years	5 (14.3)	4 (12.1)	9 (13.2)
41–50 years	12 (34.3)	13 (39.4)	25 (36.8)
51–60 years	15 (42.9)	12 (36.4)	27 (39.7)
61–70 years	3 (8.6)	3 (9.1)	6 (8.8)
Height, cm, mean (SD)	164.3 (6.6)	164.5 (6.5)	164.4 (6.5)
BMI, kg/m², mean (SD)	28.48 (5.84)	27.00 (5.73)	27.76 (5.79)
Time since diagnosis, *n* (%)			
1–3 years	14 (40.0)	11 (33.3)	25 (36.8)
3–6 years	7 (20.0)	8 (24.2)	15 (22.1)
6–10 years	4 (11.4)	8 (24.2)	12 (17.6)
10–20 years	9 (25.7)	5 (15.2)	14 (20.6)
> 20 years	1 (2.9)	1 (3.0)	2 (2.9)
Regular medication use, *n* (%)	25 (71.4)	25 (75.8)	50 (73.5)
Musculoskeletal pain affecting the back, neck, or knees, *n* (%)	33 (94.3)	32 (97.0)	65 (95.6)
Irritable bowel syndrome, *n* (%)	21 (60.0)	21 (63.6)	42 (61.8)
Depression, *n* (%)	24 (68.6)	18 (54.5)	42 (61.8)
Chronic fatigue syndrome, *n* (%)	20 (57.1)	19 (57.6)	39 (57.4)

BMI: body mass index; SD: standard deviation.

Intervention adherence was 87.5%, corresponding to a mean of 63.0 (SD 5.6) of the 72 scheduled sessions per participant. The median number of completed sessions was 63 (IQR 58–68; range 54–72), and 3 participants (8.6%) completed all scheduled classes. The adherence estimate included both attendance at live supervised sessions and self-reported completion of recorded make-up sessions when live participation was not possible. No AEs or SAEs were reported in either group during the 6-month study period.

FIQR and PSQI data were complete for all participants at all assessment time points. DASS-42 non-response occurred only in the intervention group (*n* = 8 at 3 months, and *n* = 13 at 6 months); therefore, longitudinal DASS-42 analyses included 55 complete cases (intervention, *n* = 22; control, *n* = 33).

### Outcome measures and between-group comparisons

All outcome measures demonstrated acceptable-to-excellent internal consistency across assessment points (Table II). No significant between-group differences were observed at baseline for FIQR, PSQI, depression, anxiety, or stress (all *p* > 0.05) (Table III).

**Table II:** Internal consistency and descriptive statistics of outcome measures across assessment time points

Outcome measure	Baseline	3 months	6 months
FIQR			
Cronbach’s α	0.922	0.930	0.925
Total score, mean (SD)	59.90 (16.60)	57.08 (16.73)	55.29 (16.93)
Function	15.90 (6.67)	15.21 (6.50)	14.46 (6.63)
Overall impact	11.91 (5.08)	11.13 (5.16)	10.87 (5.18)
Symptoms	32.10 (7.57)	30.74 (7.58)	29.96 (7.62)
PSQI			
Cronbach’s α	0.727	0.725	0.726
Global score, mean (SD)	9.35 (3.70)	8.87 (3.74)	8.57 (3.58)
DASS-42 Depression			
Cronbach’s α	0.952	0.964	0.960
Mean (SD)	16.59 (11.71)	14.48 (11.13)	14.56 (11.62)
DASS-42 Anxiety			
Cronbach’s α	0.919	0.899	0.880
Mean (SD)	13.37 (9.19)	13.25 (9.17)	13.20 (9.36)
DASS-42 Stress			
Cronbach’s α	0.951	0.944	0.940
Mean (SD)	19.99 (9.76)	19.77 (10.32)	19.20 (10.61)

FIQR and PSQI descriptive statistics are based on all randomized participants (*n* = 68) at all assessment time points. Depression Anxiety Stress Scales-42 (DASS-42) descriptive statistics at 3 and 6 months are based on all participants with available data at each assessment (3 months: *n* = 60; 6 months: *n* = 55). Cronbach’s α values indicate the internal consistency of each questionnaire at the corresponding assessment time point.

FIQR: Revised Fibromyalgia Impact Questionnaire; PSQI: Pittsburgh Sleep Quality Index; SD: standard deviation.

**Table III:** Between-group comparisons of outcome measures at baseline, 3 months, and 6 months

Outcome	Time	Intervention, *n*	Median (Q1-Q3)	Control, *n*	Median(Q1–Q3)	Mean difference, intervention−control (95% CI)	*U*	*z*	*p*	*r*
FIQR	Baseline	35	59.17 (45.92–69.42)	33	61.83 (51.00–71.00)		520.00	−0.70	0.484	0.08
	3 months	35	52.33 (42.50–64.75)	33	61.00 (53.50–70.67)	−8.98 (−16.96 to −1.01)	381.50	−2.40	0.016	0.29
	6 months	35	50.67 (41.17–58.33)	33	62.00 (52.83–72.50)	−11.81 (−19.61 to −4.02)	319.50	−3.16	0.002	0.38
PSQI	Baseline	35	9.00 (7.00–11.50)	33	10.00 (6.00–12.00)		575.00	−0.02	0.980	< 0.01
	3 months	35	8.00 (6.00–10.00)	33	10.00 (5.00–12.00)	−0.37 (−2.20 to 1.45)	530.50	−0.57	0.567	0.07
	6 months	35	8.00 (6.00–10.00)	33	10.00 (6.00–11.00)	−0.89 (−2.63 to 0.85)	509.00	−0.84	0.402	0.10
Depression	Baseline	35	14.00 (7.00–25.50)	33	13.00 (7.00–22.00)		592.50	0.18	0.859	0.02
	3 months	27	8.00 (5.00–14.00)	33	14.00 (7.00–24.00)	−6.40 (−11.91 to −0.89)	312.50	−1.97	0.049	0.25
	6 months	22	5.50 (3.00–15.50)	33	14.00 (7.00–23.00)	−6.70 (−12.71 to −0.68)	234.50	−2.20	0.028	0.30
Anxiety	Baseline	35	12.00 (5.00–19.50)	33	13.00 (7.00–16.00)		535.50	−0.51	0.610	0.06
	3 months	27	9.00 (4.50–15.50)	33	13.00 (7.00–17.00)	−2.81 (−7.52 to 1.89)	357.50	−1.30	0.193	0.17
	6 months	22	9.00 (3.25–16.25)	33	14.00 (7.00–17.00)	−3.59 (−8.70 to 1.52)	277.00	−1.47	0.141	0.20
Stress	Baseline	35	17.00 (13.00–29.50)	33	17.00 (14.00–29.00)		592.00	0.17	0.863	0.02
	3 months	27	15.00 (9.50–27.00)	33	18.00 (14.00–30.00)	−3.21 (−8.63 to 2.21)	355.50	−1.33	0.183	0.17
	6 months	22	13.50 (6.00–25.25)	33	17.00 (14.00–30.00)	−4.20 (−10.34 to 1.95)	273.00	−1.54	0.124	0.21

Outcome scores are presented as median (Q1–Q3). Between-group comparisons were performed using the Mann–Whitney *U* test. *U* = Mann–Whitney test statistic; *z* = standardized test statistic; *r* = effect size calculated as $\left|z\right|/\sqrt{N}$, where *N* represents the number of valid observations for each comparison. All *p*-values are two-sided. Lower FIQR, PSQI, and DASS-42 scores indicate more favourable outcomes. Negative mean differences favour the intervention group.

DASS-42: Depression Anxiety Stress Scales-42; FIQR: Revised Fibromyalgia Impact Questionnaire; PSQI: Pittsburgh Sleep Quality Index; Q1: first quartile; Q3: third quartile.

At 3 months, the intervention group demonstrated significantly lower FIQR scores than the control group, with an unadjusted between-group mean difference of –8.98 points (95% CI –16.96 to –1.01; *p* = 0.016). Depression scores were also significantly lower in the intervention group, with a mean difference of –6.40 points (95% CI –11.91 to –0.89; *p* = 0.049). No statistically significant between-group differences were observed for PSQI, anxiety, or stress (see Table III).

At 6 months, the intervention group continued to demonstrate significantly lower FIQR scores than the control group, with an unadjusted between-group mean difference of -11.81 points (95% CI –19.61 to –4.02; *p* = 0.002). Depression scores also remained significantly lower in the intervention group, with a mean difference of -6.70 points (95% CI –12.71 to –0.68; *p* = 0.028). Between-group differences for PSQI, anxiety, and stress remained non-significant (see Table III).

### Longitudinal intervention effects

*Fibromyalgia impact.* A significant group × time interaction was observed for FIQR, *F*(1.79, 118.26) = 10.08, *p* < 0.001, partial η² = 0.132, alongside significant group and time effects (Table IV). FIQR scores progressively decreased in the intervention group while remaining stable in controls (Fig. 1).

**Table IV:** Mixed repeated-measures ANOVA for longitudinal changes in study outcomes

Outcome	Effect	df	F	*p*-value	Partial η²p
FIQR	Group	1, 66	4.12	0.047	0.059
	Time†	1.79, 118.26	8.46	< 0.001	0.114
	Group × time†	1.79, 118.26	10.08	< 0.001	0.132
PSQI	Group	1, 66	0.19	0.662	0.003
	Time	2, 132	25.12	< 0.001	0.276
	Group × time	2, 132	9.82	< 0.001	0.129
Depression	Group	1, 53	2.92	0.093	0.052
	Time†	1.44, 76.47	0.80	0.415	0.015
	Group × time†	1.44, 76.47	7.85	0.002	0.129
Anxiety	Group	1, 53	1.79	0.187	0.033
	Time†	1.69, 89.52	1.32	0.270	0.024
	Group × time†	1.69, 89.52	0.30	0.701	0.006
Stress	Group	1, 53	1.17	0.284	0.022
	Time	2, 106	0.63	0.534	0.012
	Group × time	2, 106	3.68	0.028	0.065

Mixed repeated-measures analysis of variance (mixed ANOVA) was performed with time (baseline, 3 months, and 6 months) as the within-subject factor and group (intervention vs control) as the between-subject factor. Partial η²p is reported as the measure of effect size.

† Greenhouse–Geisser correction was applied where the assumption of sphericity was violated. FIQR and PSQI analyses included all randomized participants (*n* = 68). DASS-42 longitudinal analyses were based on 55 participants with complete data across all 3 assessment points (intervention, *n* = 22; control, *n* = 33).

ANOVA: analysis of variance; DASS-42: Depression Anxiety Stress Scales-42; FIQR: Revised Fibromyalgia Impact Questionnaire; PSQI: Pittsburgh Sleep Quality Index.

**Fig. 1:**
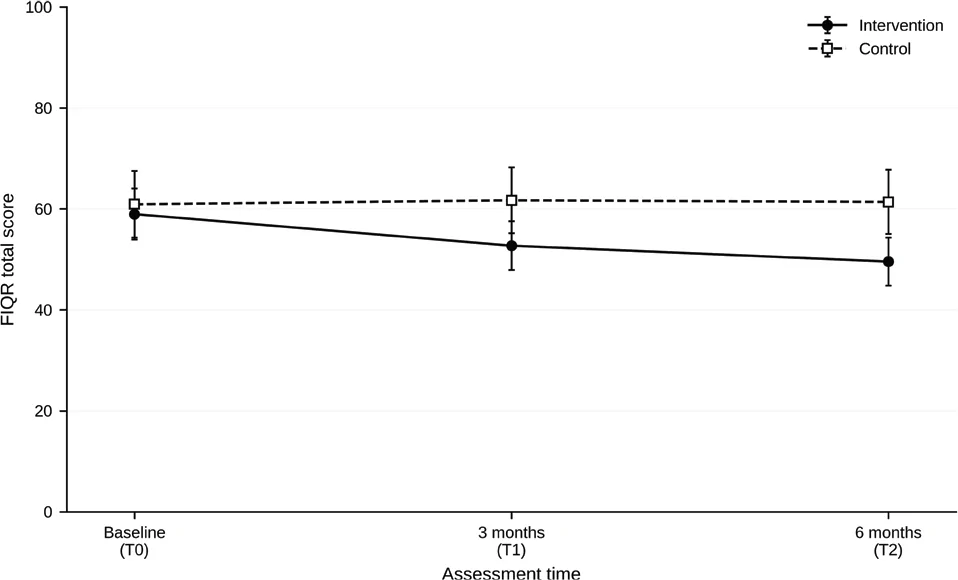
Changes in Revised Fibromyalgia Impact Questionnaire (FIQR) scores in the intervention and control groups across baseline (T0), 3-month (T1), and 6-month (T2) assessments. Values are observed means with 95% confidence intervals (CIs).

*Sleep quality.* PSQI showed a significant group × time interaction, *F*(2, 132) = 9.82, *p* < 0.001, partial η² = 0.129, alongside a significant time effect but no group effect (see Table IV). Sleep quality improved more in the intervention group, although mean PSQI scores remained above the conventional threshold for poor sleep quality at 6 months (Fig. 2).

**Fig. 2:**
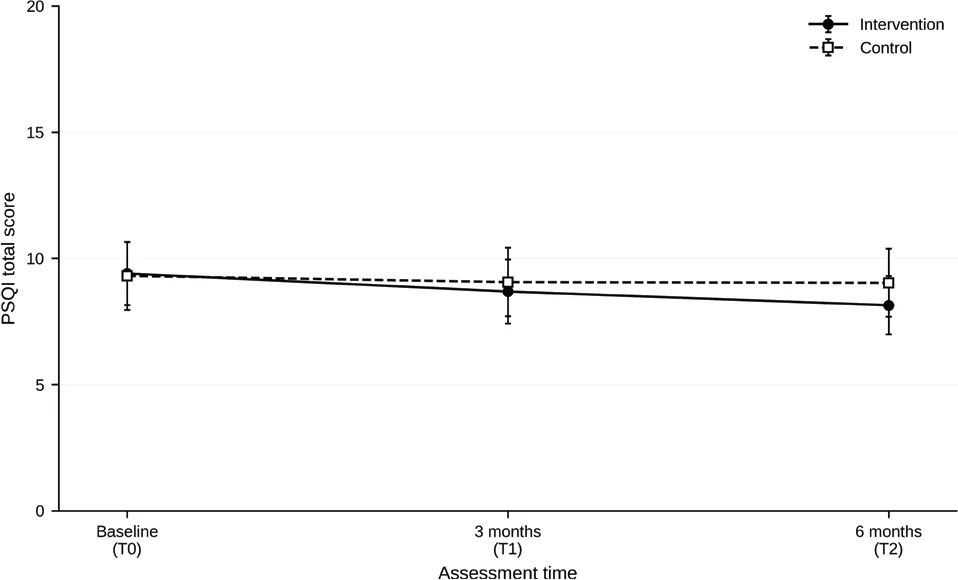
Changes in Pittsburgh Sleep Quality Index (PSQI) scores in the intervention and control groups across baseline (T0), 3-month (T1), and 6-month (T2) assessments. Values are observed means with 95% confidence intervals (CIs).

*Depression.* Depression showed a significant group × time interaction, *F*(1.44, 76.47) = 7.85, *p* = 0.002, partial η² = 0.129, whereas the group and time main effects were non-significant (see Table IV; Fig. 3).

**Fig. 3:**
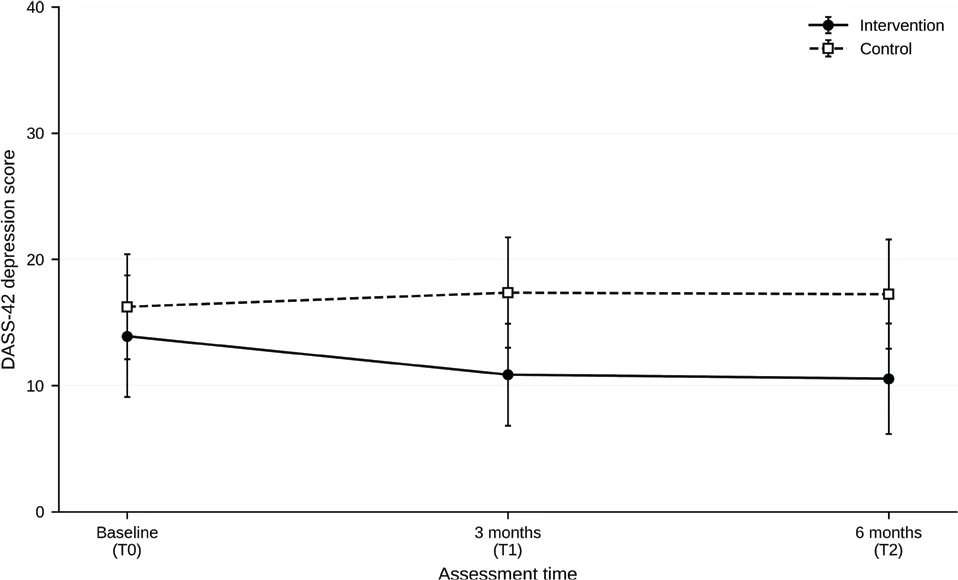
Changes in Depression Anxiety Stress Scales-42 (DASS-42) depression scores in the intervention and control groups across baseline (T0), 3-month (T1), and 6-month (T2) assessments. Values are observed means with 95% confidence intervals (CIs).

*Anxiety.* No significant group × time interaction was observed for anxiety, *F*(1.69, 89.52) = 0.30, *p* = 0.701, partial η² = 0.006; group and time main effects were also non-significant (see Table IV; Fig. 4).

**Fig. 4:**
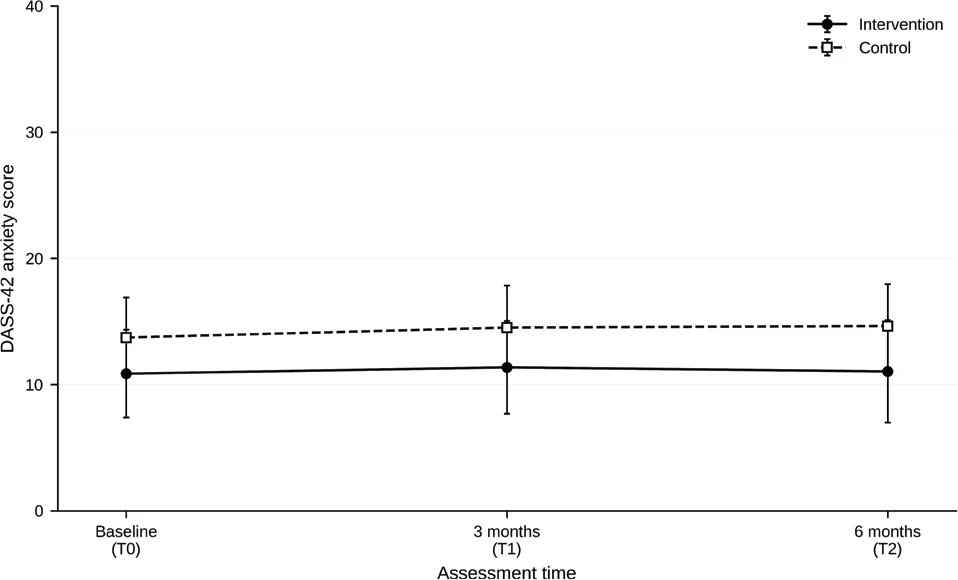
Changes in Depression Anxiety Stress Scales-42 (DASS-42) anxiety scores in the intervention and control groups across baseline (T0), 3-month (T1), and 6-month (T2) assessments. Values are observed means with 95% confidence intervals (CIs).

*Stress.* Stress showed a significant group × time interaction, *F*(2, 106) = 3.68, *p* = 0.028, partial η² = 0.065, whereas group and time main effects were non-significant (see Table IV). The intervention group demonstrated a more favourable longitudinal trend (Fig. 5).

**Fig. 5:**
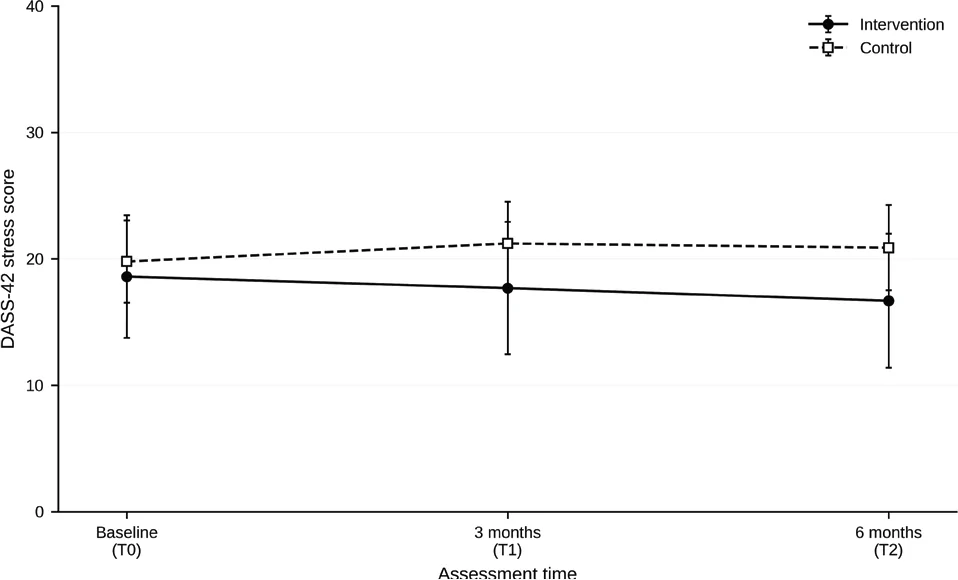
Changes in Depression Anxiety Stress Scales-42 (DASS-42) stress scores in the intervention and control groups across baseline (T0), 3-month (T1), and 6-month (T2) assessments. Values are observed means with 95% confidence intervals (CIs).

### Associations among outcomes

FIQR, PSQI, depression, anxiety, and stress were positively correlated across all assessment points (Table V). At baseline, FIQR was moderately associated with PSQI (*r*_s_ = 0.444), depression (*r*_s_ = 0.468), anxiety (*r*_s_ = 0.494), and stress (*r*_s_ = 0.370). These correlations persisted at 6 months (*r*_s_ = 0.385, *r*_s_ = 0.544, *r*_s_ = 0.477, *r*_s_ = 0.404), while strong correlations were consistently observed between depression and anxiety (*r*_s_ = 0.710), depression and stress (*r*_s_ = 0.707), and anxiety and stress (*r*_s_ = 0.734). These findings should not be interpreted as evidence of causal relationships, because the correlations were cross-sectional at each assessment.

**Table V:** Spearman correlations among FM impact, sleep quality, and psychological outcomes at baseline, 3 months, and 6 months

Variable	1	2	3	4	5
Panel A. Baseline (T0)					
1. FIQR	—				
2. PSQI	0.444***	—			
3. Depression	0.468***	0.369**	—		
4. Anxiety	0.494***	0.483***	0.731***	—	
5. Stress	0.370**	0.343**	0.615***	0.732***	—
Panel B. 3 months (T1)					
1. FIQR	—				
2. PSQI	0.317**	—			
3. Depression	0.418***	0.478***	—		
4. Anxiety	0.385**	0.411***	0.665***	—	
5. Stress	0.325*	0.389***	0.657***	0.663***	—
Panel C. 6 months (T2)					
1. FIQR	—				
2. PSQI	0.385***	—			
3. Depression	0.544***	0.410**	—		
4. Anxiety	0.477***	0.415**	0.710***	—	
5. Stress	0.404**	0.343**	0.707***	0.734***	—

Values are Spearman’s rank correlation coefficients (*r*_s_), calculated using pairwise complete observations. At baseline, *n* = 68 for all correlations. At 3 months, *n* = 68 for the FIQR–PSQI correlation and *n* = 60 for correlations involving depression, anxiety, or stress. At 6 months, *n* = 68 for the FIQR–PSQI correlation and *n* = 55 for correlations involving depression, anxiety, or stress.

* *p* < 0.05.

** *p* < 0.01.

*** *p* < 0.001.

FIQR: Revised Fibromyalgia Impact Questionnaire; PSQI: Pittsburgh Sleep Quality Index.

## DISCUSSION

This RCT demonstrates that a 6-month supervised multicomponent telerehabilitation programme combining Pilates-based therapeutic exercise with SMR produces more favourable longitudinal changes in several core domains of FM compared with usual care. Significant group × time interactions emerged for overall disease impact, sleep quality, depressive symptoms, and perceived stress, whereas anxiety remained largely unchanged. In parallel, consistent associations among FM impact, sleep disturbance, and psychological distress were observed across assessment time points. These insights reinforce the multidimensional framework of FM, highlighting the intricate interplay between physical symptoms, sleep disruptions, and emotional well-being (3, 28, 29).

The progressive reduction in FIQR scores over the 6-month intervention suggests that the programme influenced overall FM burden rather than a single symptom domain. At 6 months, the unadjusted between-group mean difference in FIQR was –11.81 points (95% CI –19.61 to –4.02), with the point estimate exceeding the prespecified 8.1-point difference used for sample-size planning; however, the confidence interval included effects smaller than this threshold. Importantly, the 8.1-point value was derived from the original FIQ rather than the FIQR and should therefore not be interpreted as an established FIQR-specific MCID. Although Surendran and Mithun (30) proposed an FIQR MCID of 27.04 points (45.5% improvement) in patients with moderate-to-severe pain, this estimate was derived from a specific clinical population and has not become a universally accepted threshold. Accordingly, the magnitude of the present between-group difference should be interpreted alongside its confidence interval and the overall pattern of longitudinal effects rather than against a single definitive FIQR MCID. These findings align with systematic reviews and clinical guidelines positioning exercise-based rehabilitation as a cornerstone of FM management. Specifically, aerobic and resistance training – whether stand-alone or part of multicomponent exercise programmes – can relieve overall symptom burden while improving both function and quality of life, as reflected in FIQ/FIQR outcomes (6, 31–33). This interpretation is also consistent with evidence supporting multicomponent treatment frameworks in FM (34, 35).

Regarding sleep quality, the intervention group showed a more favourable longitudinal trajectory. This improvement is well documented, as structured exercise typically upgrades subjective sleep in FM, though efficacy depends on participant profiles and specific protocol parameters (31, 36). Crucially, 6-month PSQI scores still exceeded the threshold for poor sleep, indicating that the programme alleviated rather than normalized sleep patterns. Although these data demonstrate a clear therapeutic benefit, they simultaneously underscore the deeply entrenched nature of sleep disturbances in this population.

Depressive symptoms and perceived stress also tracked more favourably over time in the intervention group, reinforcing evidence that therapeutic exercise supports psychological well-being in FM (31). In contrast, anxiety levels showed no significant group-by-time interaction. This differential outcome highlights that psychological comorbidities do not respond uniformly; anxiety in FM may reflect additional psychological and behavioural factors that were not directly addressed by our programme. Accordingly, adjunctive psychological or behavioural strategies may be required for patients with substantial anxiety symptoms.

Correlation analyses reinforce this view. Throughout the study, overall FM impact and sleep quality were consistently coupled with emotional distress – including depression, anxiety, and stress scales – indicating that these domains are closely interconnected. These associations mirror the multidimensional nature of FM, where somatic symptoms and sleep disruptions interact dynamically with psychological well-being as mutually reinforcing outcomes (3, 28, 29). Ultimately, these findings underscore the clinical necessity of adopting a multidimensional assessment strategy rather than focusing on isolated symptoms.

Our results expand the established literature on Pilates-based exercise for FM. While historical data document significant benefits regarding pain attenuation, functional capacity, and quality of life within this patient cohort (7, 8, 37), emerging randomized trials further validate the efficacy of digital, home-based clinical Pilates specifically for women with FM (19). In our protocol, pairing Pilates with SMR – a technique known to induce acute improvements in range of motion and patient-reported outcomes (11, 12, 14, 15, 17, 38) – was intended to yield complementary benefits by combining structured, progressive movement with targeted discomfort mitigation. However, the independent contribution of either Pilates or SMR – and any synergistic interaction between them – remains unquantified, because our intervention was delivered as a fully integrated programme without separate Pilates-only or SMR-only groups. These therapeutic effects likely stem from enhanced tolerance to physical movement and progressive loading, though the precise underlying pathways were not directly evaluated.

The mode of delivery may also have supported sustained participation throughout the 6-month programme. Attendance reached 87.5% (mean 63.0 [SD 5.6] of 72 scheduled sessions) – a notable adherence level given the well-documented barriers to long-term exercise engagement in FM. For context, a meta-analysis of 89 randomized trials reported an overall exercise dropout rate of 19.2%, highlighting continuous professional supervision as a key protective factor against attrition (39). Although live supervision served as the primary delivery mode, patients who missed their usual session could attend the alternative live class or use a recorded make-up option. This flexible, predominantly synchronous framework aligns with literature endorsing telerehabilitation – including virtual clinical Pilates and hybrid digital designs – as a viable strategy for exercise-based care in FM (18–21). However, the precise impact of this flexibility on overall adherence remains unquantified, because recorded-session completion was self-reported rather than objectively monitored.

### Clinical implications for rehabilitation practice

These findings suggest that prolonged, predominantly synchronous home-based telerehabilitation is feasible for adults with FM. The absence of reported AEs or SAEs over 6 months, together with high programme adherence, further supports the tolerability of this supervised home-based model. Real-time supervision allows clinicians to implement symptom-contingent exercise adaptations, while flexible scheduling and recorded make-up sessions provide practical options to sustain engagement. Although this integrated protocol combined low-load strengthening and stabilization with SMR techniques, these clinical insights reflect the multicomponent programme as a unified intervention rather than isolated modalities. Given the fluctuating nature of FM symptoms, effective rehabilitation models should prioritise individualized exercise modifications and flexible progression over rigid, predetermined training schedules.

From an implementation perspective, delivering 3 supervised sessions per week may require greater clinician time and organizational resources than lower-frequency rehabilitation models. In the present trial, 2 daily cohorts were offered primarily to provide scheduling flexibility rather than to increase the prescribed exercise dose; participants attended 1 session on each scheduled training day. In routine practice, feasibility would therefore depend on group size, staff availability, digital infrastructure, and the extent to which flexible scheduling or asynchronous make-up options can be incorporated. Group-based videoconferencing may partly offset resource demands by allowing 1 clinician to supervise multiple patients simultaneously, although formal cost-effectiveness and implementation analyses were beyond the scope of this trial.

### Study strengths, limitations, and future directions

Key strengths of this trial centre on its randomized controlled design, the 6-month duration of supervised telerehabilitation, and the novel integration of Pilates-based therapeutic exercise with SMR. Additionally, the structured translation and cross-cultural adaptation of the FIQR – supported with internal consistency across all time points – validates its utility within this Greek-speaking cohort.

Several methodological limitations should be considered. First, recruiting participants via online FM communities potentially introduced selection bias, and the predominantly female sample restricts the generalizability of our findings. Remote delivery formats are also inherently vulnerable to digital-access barriers and home-based intervention fidelity variations. Structurally, the multicomponent design prevents us from isolating individual therapeutic or delivery effects, while blinding participants and intervention providers was not feasible. Furthermore, reliance on self-reported outcomes precluded objective assessment of physical or physiological changes, while recorded make-up sessions were verified through participant self-report, introducing uncertainty into adherence estimates. Regarding the longitudinal DASS-42 analyses, our reliance on complete cases, coupled with differential missingness confined to the intervention group, may have introduced attrition-related bias and restricted statistical power. Finally, the absence of a post-intervention follow-up window limits our ability to evaluate the long-term durability of the treatment effects.

Future research should prioritize larger and more diverse cohorts, use objective clinical and adherence measures, and incorporate extended post-intervention follow-up periods. Factorial or multi-arm designs are also needed to clarify the relative contributions of individual therapeutic and delivery components, including the specific role of asynchronous make-up sessions.

In conclusion, a 6-month, supervised, home-based multicomponent telerehabilitation programme pairing Pilates-based therapeutic exercise with SMR resulted in more favourable longitudinal changes across key FM domains – specifically disease impact, sleep quality, depressive symptoms, and perceived stress – relative to usual care. Conversely, anxiety levels appear to remain largely unaltered by this format over time. High programme adherence and the absence of reported AEs or SAEs in either group support the feasibility and tolerability of this predominantly synchronous digital model. Taken together, our outcomes highlight the therapeutic value of long-term, multimodal telerehabilitation combining Pilates-based therapeutic exercise and SMR for adults with FM, reinforcing the necessity of addressing their diverse, interconnected symptom profiles.

## Supplementary Material


