# Supplementary material for: SIX-MONTH SUPERVISED TELEREHABILITATION COMBINING PILATES-BASED THERAPEUTIC EXERCISE AND SELF-MYOFASCIAL RELEASE IN ADULTS WITH FIBROMYALGIA: A RANDOMIZED CONTROLLED TRIAL
Source: J Rehabil Med. 2026 Oct 13;58:46786. doi: 10.2340/jrm.v58.46786 (PMC13673445; doi:10.2340/jrm.v58.46786)

Fig. S1. CONSORT 2025 flow diagram of participant recruitment, allocation, follow-up, and analysis.

Note. All 68 randomised participants remained in the study through 6 months. FIQR and PSQI were complete at all assessment points. DASS-42 questionnaire non-response occurred only in the intervention group (8 at 3 months; 13 at 6 months); the 8 participants missing at 3 months were among the 13 missing at 6 months.

CONSORT: Consolidated Standards of Reporting Trials; DASS-42: Depression Anxiety Stress Scales-42; FIQR: Revised Fibromyalgia Impact Questionnaire; PSQI: Pittsburgh Sleep Quality Index.

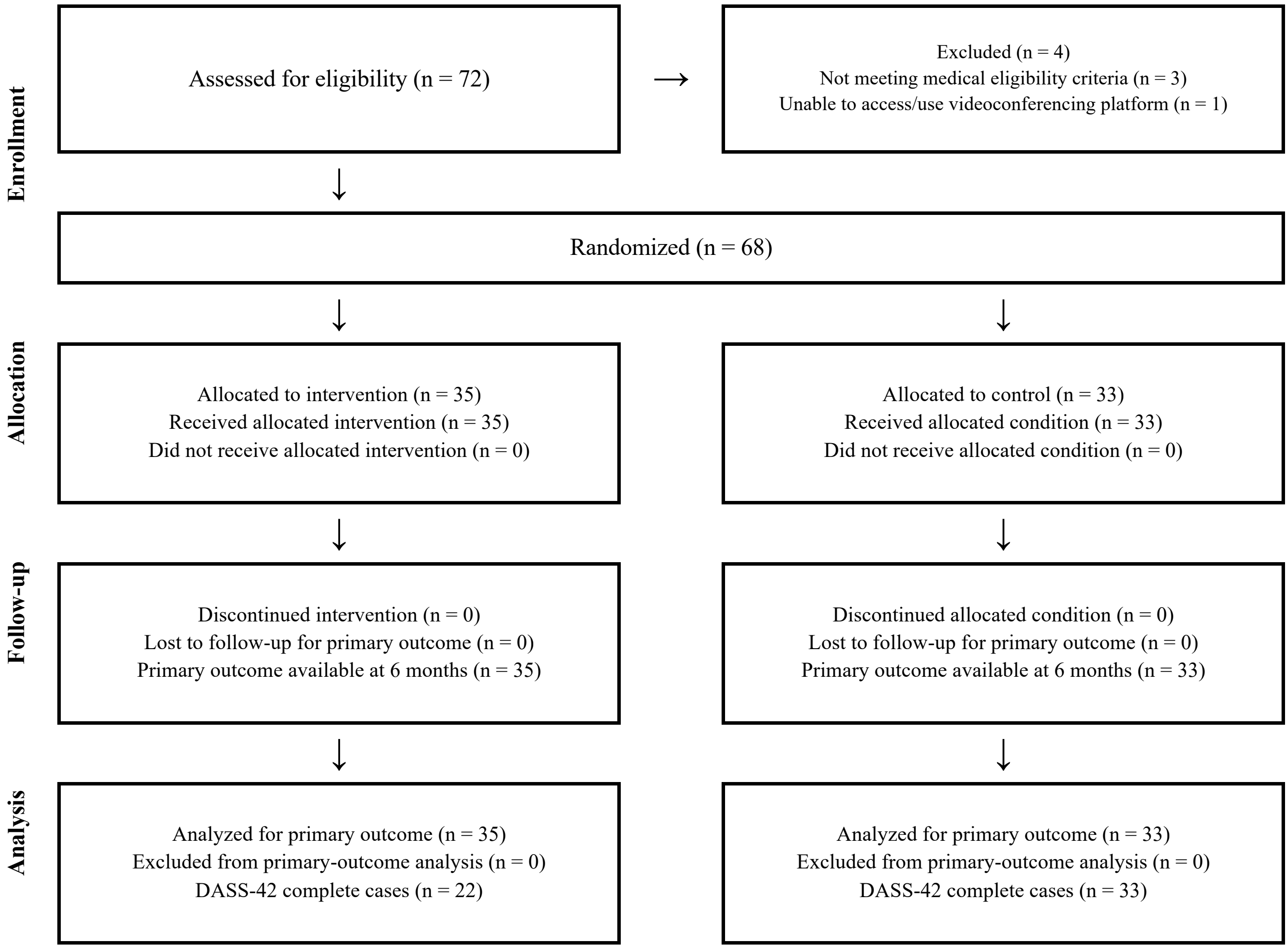

Supplement: Supplementary file 1 [file JRM-58-46786-s1.pdf]
